# Extracts from an Essay on Diptheria and Diptheritic Affections

**Published:** 1860-12

**Authors:** A. Jacobi


### EXTRACTS FROM AN ESSAY ON DIPHTHERIA AND DIPHTHERITIC AFFECTIONS.

BY A. JACOBI, M. D.

(Concluded.)

Our knowledge of the pathological anatomy of diphtheria is very defective, especially in regard to such alterations as take place in the blood and general system. The microscopical appearance of the pseudo-membranes has been described in our remarks on the nature of the disease; oidium albicans, and leptothrix buccalis have been found by some writers, and have even been considered by them to be the cause of contagious transmission of the affection; we have not, however, been able to identify them in those few microscopical examinations we have made; and we believe that those microscopical fungi are but accidental occurrences in the exudation. We have further described the appearance of the mucous membranes as to whether the exudation was but a covering to its substance, or imbedded in its tissue and leaving more or less deep ulcerations. Pneumonia, bronchitis, and the results of complications of diphtheria were not unfrequent in those fifteen or sixteen cases where we were allowed to make a post mortem examination. The skin would sometimes present the appearance of furfuraceous desquamation on some parts of the body, as it will be found in a large number of diseases attended by high fever and rapid diminution of the subcutaneous tissue. Petechiæ were found in a few instances, generally most in the præcordial region, hypogastric region, and on the thighs. The submaxillary and cervical glands, with the surrounding tissue, were generally swelled, but not to such an extent as might be expected from the swelling during life; the tonsils were either swelled, not hyperæmic, or covered with deep ulcerations, or again consisting, to a great extent, of a grey, fibrous mass; uvula ulcerated, sometimes much diminished in size; liver and spleen, in a few cases, greatly hyperæmic in spite of general anæmia; in one instance there were apoplectic clots in the tissue of the spleen; in others they were as anæmic as the body in general. The blood was generally thin and liquid, and several times of a dark, scorbutic color; the meningeal blood-vessels were distended with blood, undoubtedly a consequence of moribund condition. In the case of death from uræmic convulsions, in Hoboken, a small vein near the left sinus transversus was ruptured, and a small clot of blood presented itself outside the vessel. Thus the results of post mortem examinations are certainly few; there is nothing in them characteristic of diphtheria, except the local alterations; and in this respect diphtheria takes part in the general nature and anatomical mystery of the zymotic diseases as a class.

The diagnosis of diphtheria, and particularly of diphtheritic affections of the pharynx, ought never to be difficult. At all events, it can be made with less difficulty than scarlatina, which will not unfrequently be recognized from its consecutive diseases only; for there is one pathognomic symptom which will never fail to give certainty, viz., the pseudo membrane. All the other symptoms, as headache and earache, submaxillary and cervical adenitis, erythematous or œdematous swelling of the fauces, fever, convulsions, toxæmia, and prostration, foul smell from the mouth and nose, albuminuria and hemorrhage, severe though they may be, are not pathognomonic. Each of these symptoms may either be produced by other diseases, amydalitis or general pharyngytis, any feverish disease, particularly scarlatina or other acute exanthems, gangrenous sore throat, or meningitis; or they may be absent in a case of genuine diphtheritic affection. The difference, as to severity and danger, in diphtheritic affections is at least as great as in any other epidemic malady; and many cases have such an innocent appearance that authors have been induced to comprehend them under the name of herpetic angina. As to this we have stated our reasons for considering them as mild forms of diphtheria, which, however, may lead to very severe consequences; there is in them a local diphtherite with no fever or only a slight febrile attack with intermittent character. Nasal discharge, and foul smell from the nose and mouth are not at all required to ensure the diagnosis, nor is any symptom of the same importance as the exuded membrane. It will not be difficult to distinguish the diphtheritic membrane. It will not be difficult to distinguish the diphtheritic membrane from gangrenous pharyngitis; but it must be well borne in mind, that local gangrene may be a complication of diphtheria. Even Bretonneau has observed, that diphtheritic membranes would sometimes cover the exulcerations produced by pultaceous cynanche. The differential differential diagnosis from scarlatina will, in some instances, cause difficulty for several reasons. Scarlatina is frequently complicated with diphtheria; thus, wherever the eruption is of no account or escapes the attention of the physician, a mistake is easily made. Secondly, albuminuria is common to both, and prrhaps there is no other difference between the two except this, in many instances, that the urine of scarlet fever patients will more generally show casts. And finally there is, too, an erythematous eruption of scarlatina. Some writers, like Dr. Peter, take such cases to be scarlet fever complicated with diphtheria; such an one of the two maladies as is produced by a greater epidemic influence exhibiting the principal symptoms. We feel no inclination to agree with this opinion; for such cases will sometimes turn out as diphtheria, from subsequent symptoms, as, for instance, paralysis; no scarlatinous desquamation has ever been observed by us, and the little furfuraceous desquamation that sometimes will take place, has, in some instances, been found where no erythematous eruption is not an absolutely uncommon occurrence in any of the febrile diseases of infancy. We finally add, that we have observed diphtheria in individuals, and even returning for the second time, who had suffered from scarlatina before. This remark we make in regard to those who are inclined to recognize an indentity between scarlatina and diphtheria.

One case under our observation offered a remarkable point as to diagnosis. A man suffered from what appeared to us to be pharyngeal diphtheria, with fever, etc., for some days before he was taken into the Jews' Hospital; he grew anæmic and prostrated, but the membranes did not take very long to disappear from the soft palate and uvula, under a simple diphtheritic treatment. Something like a fortnight afterwards the uvula and soft palate again exhibited a grey coating, which soon, in the course of a few days, increased consderably in size, and showed the symptoms of syphilitic ulcerations. The patient denied ever having suffered from chancre, nor could we discover any marks. Our antidiphtheritic treatment was continued for about six days, until the soft palate was perforated. Feeling sure, then, that we had to deal with secondary syphilis, probably brought on prematurely by the preceeding diphtheritic affection and œdematous swelling of the parts, we commenced a mercurial treatment; the patient confessed as to his having had syphhilis, but it was then too late to save all of his soft palate.

The prognosis depends on the condition of the child preceding the attack, on its age and general strength, on the character of the affection, and on the absence or presence of dangerous complications. Children will die in a larger proportion than adults; these being affected in a larger number with diphtheria than with scarlatina will, as a general rule, take effect once during the life of a patient, while diphtheria has a great tendency to return. Anæmic and sickly children are in great danger under all circumstances; cutaneous diphtheria will prove fatal in many instances during the first year where cutaneous eruptions are common occurrences. The extension of membranous exudation in the pharynx, etc., does not always produce a proportionate malignity, except in small children where the breathing is particularly effected through the nose, and even a slight obstruction of its cavities and moderate swelling of the pharynx is able to prevent decarbonization of the blood. Scarlatina, measles, noma, gangrenous sore-throat, large swelling of the submaxillary and cervical glands, high fever extensive pneumonia and bronchitis give no favorable prognosis. But the average mortality is not so great as might be inferred from the reports published on some epidemics. The cases of mild form and average severity are the large majority, and will recover with a rational treatment. Severe cases often perish in spite of any thing that may be done to relieve them. Cases that set in with a high fever, considerable adenitis, cerebral symptoms, intense head-ache and ear-ache, and convulsions, small, frequent pulse, and a foul smell from the nostrils and mouth, must be considered as extremely dangerous. Hemorrhages, at any place, and of any kind, must be considered as a severe complication, as they indicate a deep-seated alteration of the blood; we have observed hemorrhages from the nose (right nostril) and intestines, and petechiæ. They must be considered as of equal importance to those occurring during or after scarlatina, erysipelas, etc., even in some of those cases where the nasal hemorrhage is produced, as causa proxima, by exulcerations of the nasal mucous membrane. Cases of average severity will take from five or six to ten and twenty days to recover: severe cases, unless they prove fatal during the first period, may take several weeks or months. But the large majority, according to our experience, ultimately recover. Of about five hundred cases, we believe we have lost not more than thirty, but we have seen very protracted convalescences in most of them, depending on the prostration of the nervous power and the anæmic condition of the patients. The causes of death are of various nature. Sometimes it is the dissolution of the blood, the exceedingly high fever, cerebral symptoms at the first onset or towards the end, exhaustion from want of food and absence of digestive power, general nervous prostration, protracted anæmia, or suffocation from obstruction of the respiratory organs.

We need not add that the prognosis becomes more unfavorable than it will be under other circumstances, from the fact that the disease is very apt to return. A second attack in a child exhausted by a preceding one will readily prove fatal. Even laryngeal diphtheria has been observed to recur, although we have never seen an instance of croup twice occurring in the same individual. Guersant performed tracheotomy twice in each of two children, after intervals between the two attacks of eleven and twenty-one months. A few other cases of the same kind have been reported.

One of the most remarkable consequences of diphtheria is total or partial paralysis or paresis. Paralysis of the velum palati will, in many cases, be observed during the course of the exudative process and œdematous swelling, and in this case must be taken as directly depending on the natural alteration of the tissue. The voice will have a nasal twang, and food, particularly of the liquid kind, will regurgitate through the nose, but in other instances, it will not make its appearance before the process is all over, and the patient, although still anæmic, on a fair way to convalescence. We saw, this week, a little boy of three years, who, for the last four months, has had this nasal twang and suffered from regurgitations of liquid food, after having gone through a slight diphtheritic affection of the pharynx, in the first half of January. The same remarks are applicable to other forms of paralysis, as strabismus, of which we have observed several cases in children. We may here state that we have seen no other forms than those two enumerated in infancy. In adults we have observed several instances of general paresis of either the motory or the sensory nerves, sometimes the two together. The lower extremities will be the first affected, and afterwards the sexual parts, upper extremities, hearing, taste, smell, and sight will be involved in the general affection. The motory nerves were mostly affected, physical exertion being extremely difficult. The youngest individual in which we have seen these general symptoms, was a lady of seventeen years, in whom motion, sensibility, sight, voice, and intelligence suffered in an equal degree. The respiratory muscles have been reported, by authors, to have been paralyzed; we have not seen any such case, unless that reported on page 96 belonged to this class. As we had no experience whatever on this point at that time, we then directed no particular attention to this fact. The prognosis in these cases is favorable; all our paralytic patients recovered in the course of from two to five months. Even complete amaurosis, of which we have not seen a case, is reported by Maingault to have recovered in a little more than six months. A few cases of death, however, have also been related; in two instances, from suffocation produced by food getting into the larynx, the pharyngeal muscles being paralyzed.

The cause of this paralysis is somewhat obscure. It is but natural that a material alteration taking place in the soft pallate should interfere with its functions. But the majority of cases date from convalescence, will sometimes appear suddenly and sometimes are gradual in their development. It may be observed after very severe attacks of diphtheria, and again after apparently a mild form; we have stated that it followed some light cases of so-called herpetic angina; and we have above pointed to the fact, that many cases of diphtheritic paralysis were preceded by albuminuria. We do not hesitate to attribute general paralysis of this kind to the want of sufficient nervous power, produced by the diphtheritic hydræma (which, too, we consider in these cases as the cause of albuminuria.) Local paralysis, we are inclined, from physiological reasons, to attribute to local extravasation or exudation, the last cause of which must also be sought for in general hydræmia and facilitated transudation. We have seen one instance of loss of hair during convalesence.

The diagnosis of diphtheritic paralysis is made sure by the history of the case, and by the difference from other forms of paralysis. Paralysis from myelitis begins with clonic and tonic convulsions, and descends slowly, and the pharynx suffers last, whereas diphtheritic paralysis shows the affection of the pharynx and the power of speaking at first. Galvanic contractility is also intact (which is not the case in spinal paralysis); and paralysis, according to what has been observed in other epidemics, has a great tendency to localization and diphtheria.

As to the treatment of diphtheria and diphtheritic affections, we venture upon the following remarks:—This disease which has been the subject of the foregoing exposition, has been shown to exhibit prominent symptoms of two different kinds, viz: local and general. Thus the treatment, where any is required, has to fulfill two distinct indications. We say, wherever it is required, for experience shows that a number of cases will get well without any treatment whatsoever, so that it is not a very unfrequent occurrence to meet with light tonsillar diphtherite without fever, or any other dangerous symptom, in children to which we are called for some other complaint. In such cases light fever may have been present, and large membranes have been exuded on or into the mucous membrane of the pharynx, and nevertheless the whole course of the disease has passed unheaded and untreated. Such an occurrence is certainly not exceptional, for the same is true of other diseases, particularly those of zymotic origin. The majority of zymotic diseases require little or no medical treatment at all, especially those running their course in a distinctly typical manner. As to diphtheria, we have even made experiments, showing that mild cases will get well wihout treatment. But we think it more dangerous in diphtheria, than in other zymotic diseases, to abstain from treatment altogether, for three reasons:—Diphtheria is not a typical malady, but has a great tendency to returu; it is more of an adynamic character than any other; and, finally, by the thick and extensive exudations in the pharynx and on the adjoining parts, it is apt to produce serious troubles, by mechanical encumbrances to deglutition and respiration.

The local treatment conists of cauterization of the membranes and surrounding parts with the solid nitrate of silver, or with strong or mild solutions of the same salt in water ( ʒ ss-j. : ℥ j.) ; of gargles, consisting of solutions of (or applying in substance) astringents, such as tannic acid, alum, sulphate of zinc, or claret wine ; in gargling with, or applying, such medidinal agents as are known to have some effect on the constitution and tissue of the pseudo-membranes, as chloride of potassium, chlorates of potassa and soda, diluted or concentrated nitric or muriatic acids, liqour of sesquichloride of iron, etc. Astringents will prevent maceration, render the exudation dry and hard, and alter the consistency of the surrounding hyperæmic and œdematous tissue. It will thus prevent, sometimes, the extension of pseudo-membranes to the neighborhood of the parts already affected, and in some cases may accelerate the expulsion of the membrane as a whole. We have thus seen the best effects from tannic acid, either applied directly to the parts by means of a curved whalebone probang, or dissolved in water so as to have a gargle, ( ʒ ssji ; ℥ j. Of the tinct. sesquichlor. iron we have seen no particular effect.

Cauterizations with nitrate of silver we have found to be generally of very little use when applied to the pharynx. Its effect is superficial only ; it will form a scurf, but will destroy nothing. Destruction of the parts cannot be effected except by forcing the caustic into and below the membrane ; this can seldom be done in the pharynx of children, and for this reason cauterization is unavailing at this point, but will prove beneficial, we believe, by confining the process of exudation to its original locality. In cutaneous diphtheria cauterization may be exercised to its full extent, but as these cases are generally attended with extreme prostration, the general treatment will prove both more necessary and successful. If cauterization is to be resorted to, we generally use, and with good effect, more or less concentrated muriatic, or acetic, or nitro-muriatic acid. Where, however, cauterizations are made, great caution is necessary not to mistake afterwards the result of the caustic for pseudo-membrane. This remark is particularly applicable where nitrate of silver has been used.

In regard to the internal administration of remedies, and the general treatment of diphtheria, we have to remember that it is eminently an adynamic disease. Prostration will set in, and complete exhaustion will sometimes destroy patients in spite of the most careful treatment. As a general rule, therefore, no remedy should be administered that will increase the amount of water in the blood, accelerate its decomposition, or exhaust the nervous power. Mercury must be avoided, no blood be drawn, no vesicatories applied, in a word, no antiphlogistic treatment should be resorted to; for the superabundance of fibrine does not indicate antiphlogistic treatment, as it is a well-known fact that in extreme hydræmia the proportion of fibrine may be greatly increased. The more hydræmia is increased, the greater the proportion of fibrine. For the same reason the careless routine practice of administering large doses of alkaline remedies, carbonates, bicarbonates, nitrates, etc., of potassa and soda, for the purpose of liquifying the fibrine, must be discarded. Their effect is generally not good, and we firmly believe that the duration of convalescence will be lengthened by their free use. Trosseau is certainly right in asserting that rationalism in medicine is very apt to lead to absurdities.

Emetics must be avoided as much as possible; but they will sometimes prove necessary to remove accumulations of mucous or macerated membrane. Then ipecac is preferable to the others. In cases where diphtheria is descending into the larynx, great caution is desirable in administering emetics; for in this form of croup exhaustion will sometimes take place unexpectedly and rapidly before suffocation.

The functions of all the organs have to be kept in order; the kidneys require special attention, the greatest danger being in the interruption of their function. Spir. nitr. dulc. squill, and parsley may be administered; but neither digitalis nor iodide of potassium. The skin will generally act well with liq. acetat. ammon., alcoholic beverages, and fricsions. Regular baths, with alcoholic or aromatic admixtures, and change of air and residence will prove beneficial. Digestion must be kept as normal as circumstances will allow; tonic and stimulating diet not only allowed but insisted upon; meat, eggs, coffee, wine and brandy are recommendable.

As a general antidiphtheritic remedy, chlorate of potassa and chlorate of soda as its substitute, have earned a good reputation. Chlorate of potassa is soluble in sixteen parts of water, and is well tolerated by the stomach. There is no necessity, therefore, for preferring the soda, which is higher in price, though it has the preferrence in dissolving in three or four parts of water. We do not agree with those who deny the efficacy of this remedy, because it does not meet all emergencies. We have used it in hundreds of cases, besides its administration in stomatitis, mercurial affections of the mouth, etc., and are perfectly satisfied with the result, except in those cases which ran an extremely rapid course with such symptoms as high adynamic fever, quick and small pulse, evidence of dissolution of the blood. In such cases it is too slow in its effects. But in all those instances where a sudden and instantaneous effect is not required, and death is not imminent, it is a highly valuable remedy. But doses of a grain or two will not prove sufficient; it ought to be given in doses of from half a drachm to one and a half drachms daily, dissolved in water alone, or combined with other remedies. If possible the patient must be made to swallow the solution slowly, to have its local as well as general effect.

Acids are very beneficial agents in this disease. Diluted muriatic acid, four, six, or ten drops every hour or two hours, more in proportion to adults; or concentrated nitro-muriatic acid, two or six drops in the same intervals, will be found to act as well locally as generally, and will, besides, increase the appetite and stimulate the digestive functions. The concentrated nitro-muriatic acid we have regarded as one principal remedy in those dangerous cases described above. Good effects have, in many instances, been observed from full doses of tannic acid, ten grains to two scruples and more being given daily, dissolved in water. It has a beautiful local effect in the pharynx when swallowed slowly, and is of invaluable service in renal affections.

As a tonic, iron is highly serviceable. The preparation most in use with the English profession, and with us also, is the tincture of the sesquichloride: its astringent effect has been praised highly, perhaps too highly; its influence on the whole system in general, and sanguification in particular, is undoubted. Its dose is, according to age, from twenty drops to one and a half or two drachms daily, alone, or, as most administered, with chlorate of potassa. Combinations of these, or the two with diluted muriatic acid, or each with the acid, are of the same practical value as they are theoretically justifiable. If tannic acid and iron were to be used, in any form or manner, at the same time, they are to be kept apart, and administered at different times; the tannate of iron being indigestible, Ozanam's formula,

Bromine gt. j,
Bromine potass. gr. ij,
Aq. ℥ ij, we have tried in fifty cases, from November, 1859, to February, 1860. We have used it in much larger doses than Dr. O., from thirty to fifty drops and more daily, after we were dissatisfied with smaller ones. We think it a valuable remedy except in severe cases; we should not then rely on it; perhaps, however, we have not sufficient experience. We have a similar remark to make in regard to the tincture of iodine, with which we have had but little experience.

Of the greatest value is that powerful febrifuge, quinine. We have but seldom used it as a tonic, but generally in one or two large doses (from five to ten grains in children, or the sulphate or muriate, the latter containing more quinine) daily. We have never seen any bad effects, but have always found a great and rapid remission of the fever. If one dose was taken we ordered it in the afternoon, usually between three and five o'clock; another was sometimes taken in the morning for one or two days, until there was no necessity of administering it twice. A *conditio sine qua non* is a full dose. A child of a year or two must not have less than five grains in a daily dose; we have even given to children of two or three years, repeated doses of ten grains, and have been fully satisfied with its effect. We have in no disease observed less cerebral symptoms attributable to the effect of quinine than in diphtheria. It will prove particularly successful in such cases as are more or less complicated with acute rheumatism. In the case of a girl of seven years, whose stomach was much disordered, we resorted to subcutaneous injections of a nearly neutral solution of mur. chin, gr. iv, on two subsequent days, with excellent success.

Albuminuria requires tannic acid; we seldom give any other remedy, and warmly recommend it. The functions of the bowels, skin, etc., require the care indicated by the rules of general and special pathology. In a case of hemorrhage from and suppuration in the kidney, in sickly diphtheritic girl of three years, we thought proper to give evratrum, in order to diminish the fever, and thus, indirectly, to relieve the congestion of the kidneys; our success was complete; the child being more healthy and robust than for years.

Descending croup, during the epidemic, proved highly dangerous. Patients in some cases had not even sufficient time to die of suffocation; but perished from the exhaustion brought on by the general malady. We feel, therefore, justified in warning against a free use of emetics, as a new source of exhaustion. We have no particular remedy to recommend; the treatment should vary according to the case. But, in referring to our remarks and cases above, we feel sure that even of such cases, many will in future be saved by tracheotomy.

Hemorrhages require, locally, astringents, and the general antidiphtheritic nutrition and treatment. Wherever the seat of hemorrhage can be reached, we doubt not but the application of tannic acid, muriated tincture of iron, or, better than anything else, persulphate of iron will prove successful instantaneously.

Paralysis requires moderate, very moderate, active and passive motion. The eyes require great care and perfect quiet. Local paralysis indicates local galvanization; thus, in paralysis of the soft palate, one pole is applied to the palate, and the other to the mastoid process. In general paralysis, iron and strychnia are indicated; the latter we have used, on the recommendation of Trousseau until a slight convulsive flection, usually in the thighs first, became perceptible. Our dose was, in adults (as we have not seen children with general paralysis), from the one-eighth to the one-sixteenth of a grain, twice a day.

Submaxillary and cervical adenitis require seldom or never any kind of depletion. In two cases only, in which active inflammation seemed to take place, we have applied leeches. Our usual treatment consisted in the application of camphorated oil, or of tincture of iodine in older cases, or of the following formula:

Iodine, ʒ ij.;

Glycerine, volatile liniment, āā ℥ ss.

In the very small number of cases in which suppuration took place we recommend early incision.

Finally, in cerebral affections, we know of nothing to recommend. Convulsions in the onset of the disease will generally not prove fatal; but such as occur after exhaustion and symptoms of dissolution of the blood have taken place, will prove the prelude of death.

